# Digestion of Starch in Infancy

**Published:** 1873-04

**Authors:** 


Digestion of Starch in Infancy. (*Gaz. Med. Ital.*, November, 1872.) It has been known that the saliva of newly-born animals has not the power of transforming starch into sugar. A recent experimenter has taken the pancreas from kittens and puppies, and has ascertained that the pancreatic juice in these animals when young is, like the saliva, incapable of converting starch into sugar. The bearing of this fact on the practice of giving starchy food to very young infants is obvious.—*N. Y. Medical Journal.*

